# *Ginkgo biloba* extract EGb 761 in patients with dementia and a history of cerebral infarction—meta-analysis of pooled data from randomised clinical trials

**DOI:** 10.3389/fneur.2025.1658064

**Published:** 2026-03-13

**Authors:** Jing-Xuan Feng, Man-Qi Zheng, Xue Tian, Andrea Zimmermann, An-Xin Wang, Xia Meng

**Affiliations:** 1 National Clinical Research Center for Neurological Diseases, Capital Medical University, Beijing, China; 2 Department of Neurology, Beijing Tiantan Hospital, Capital Medical University, Beijing, China; 3 Department of Epidemiology, Beijing Neurosurgical Institute, Beijing Tiantan Hospital, Capital Medical University, Beijing, China; 4 Department of Clinical Epidemiology and Clinical Trial, Capital Medical University, Beijing, China; 5 Division of Biometry, Dr. Willmar Schwabe GmbH & Co. KG, Karlsruhe, Germany

**Keywords:** *Ginkgo biloba* leaf extract, EGb 761, dementia, cognition, stroke, cerebrovascular, pooled data analysis, clinical trials

## Abstract

**Introduction:**

*Ginkgo biloba* leaf extracts belong to the most popular herbal medicines for the treatment of neurological disorders, including Alzheimer’s disease (AD) or stroke. EGb 761, a proprietary ginkgo leaf extract, has been shown to improve brain cell energy supply, to enhance neurogenesis and neuroplasticity, to decrease blood viscosity and improve brain perfusion. Thereby it improves cognitive performance, neuropsychiatric symptoms and activities of daily living in patients with dementia or mild cognitive impairment. It has further been shown to be beneficial for patients after ischaemic stroke. Therefore, the aim of this meta-analysis was to evaluate the treatment effects of EGb 761 in patients who had developed dementia following a cerebral infarction.

**Methods:**

We performed a meta-analysis of pooled data from clinical trials with EGb 761 in mild to moderate dementia in the subgroup of patients who had a cerebral infarction. Four randomised, placebo-controlled trials with homogeneous patient selection and design were included. Previous stroke was diagnosed by neuroimaging. The analysis focused on the comparison of treatment effects in the domains of cognition, activities of daily living and global assessment.

**Results:**

The meta-analysis included data from 488 patients. Significant treatment effects of 240 mg EGb 761 daily versus placebo were found for cognition (*p* = 0.0467), activities of daily living (*p* = 0.0230), and global clinical impression (*p* = 0.0371). The rates of adverse events and adverse drug reactions in the EGb 761 group were like those in the placebo group.

**Conclusion:**

The results of our meta-analysis of patients with mild to moderate dementia who had previously had a cerebral infarction verified by neuroimaging showed statistically significant and clinically relevant benefits of EGb 761. The drug was shown to be safe and well tolerated and is a promising treatment option for patients developing dementia after cerebral infarction. Further dedicated clinical trials are needed to confirm these results.

## 1 Introduction

While the global incidence of stroke is in decline in most regions, it is rising in China. Stroke has become the primary cause of mortality and permanent disability (1), imposing a significant burden on the national healthcare system. A review of data from 1,599 hospitals in the Hospital Quality Monitoring System and Bigdata Observatory Platform for Stroke in China revealed that over 3.4 million patients with stroke were admitted to hospitals in 2020. Most cases (80% or more) were diagnosed as ischaemic stroke, 14.9% were intracerebral haemorrhages, and 3.1% were subarachnoid haemorrhages. From 2010 to 2019, there was an increase in the incidence of ischaemic stroke, while the incidence of haemorrhagic stroke decreased (1). A study conducted on the basis of data from a national hospital information system in China showed that vascular dementia (VaD) was the most prevalent form of dementia among hospitalised patients (2).

Recent study results have highlighted the significance of cognitive impairment following strokes. In a systematic review, 16 hospital-based studies with over 3,000 patients were analysed (3). The overall prevalence of post-stroke neurocognitive disorders was shown to be 53.4%. Moreover, the ICONS Study revealed that 52.1% of patients had cognitive impairment 3 months after stroke as assessed by the Montreal Cognitive Assessment (MoCA) (4). A community-based, cross-sectional study conducted in China yielded an even higher overall prevalence of 80.1% (5).

A longitudinal cohort study conducted in China (6) enrolled patients with cerebral small vessel disease, which accounts for about 50–70% of patients with vascular cognitive impairment. Study results showed a correlation between white matter hyperintensity on magnetic resonance imaging (MRI) and cognitive dysfunction. Moreover, arteriosclerotic microbleeds and abnormal reductions in cortical cerebral blood flow were identified. The authors concluded that structural brain network disruption is pivotal for the pathogenesis of cognitive decline in these patients.

*Ginkgo biloba* leaf extracts belong to the most popular herbal medicines for the treatment of neurological disorders, such as depression, Alzheimer’s disease (AD), stroke, Huntington’s disease, and Parkinson’s disease. As a mixture of multiple plant substances, the underlying molecular mechanisms are diverse, being mainly free radical scavenging, anti-oxidant activity, anti-inflammatory response, mitochondrial protection and neurotransmitter regulation (7). The effect of *Ginkgo biloba* extract on the formation of amyloid ß-oligomers as a biomarker of Mild Cognitive Impairment (MCI) was shown in a retrospective cohort study. 64 MCI patients with amyloid detection by positron emission tomography took either 240 mg *Ginkgo biloba* extract daily (*n* = 42) or reference treatments (*n* = 22). After 12 months, *Ginkgo biloba* therapy was associated with reduced amyloid ß-protein oligomerisation a well as preserved cognition and improved daily functioning (8).

EGb 761^1^ is a special dry extract manufactured from the leaves of *Ginkgo biloba* L. It was shown to enhance neurogenesis and neuroplasticity (9), to decrease blood viscosity and to improve brain perfusion (10). Clinically, EGb 761 has been shown to improve cognitive performance, neuropsychiatric symptoms and activities of daily living in patients with dementia (11) and with mild dementia (12). An updated umbrella review showed that—among other substances—*Ginkgo biloba* extract appears to be beneficial for cognitive, global performances, and activities of daily living in patients with AD (13). A systematic search for *Ginkgo biloba* extract or EGb 761 in AD and dementia yielded 15 clinical trials for synthesis. In 11 of them, *Ginkgo biloba* extract was shown to improve cognitive function, neuropsychiatric symptoms, and functional abilities in both dementia types. Significant improvements were observed in scores obtained from the Mini-Mental State Examination, Short Cognitive Performance Test, and Neuropsychiatric Inventory (14). Another systematic review investigated therapeutic strategies including *Ginkgo biloba* extracts in vascular cognitive impairment. The plant extracts showed large to moderate improvements in cognition and small to moderate improvements in functional outcomes, thus having shown the greatest cognitive and functional benefits. However, their clinical significance remains uncertain, and certainty of evidence is overall low due to significant heterogeneity (15).

In addition to its favourable impact on cognition, Ginkgo extract has been demonstrated to confer further benefits in patients following cerebral infarction. A controlled clinical study conducted in China demonstrated that EGb 761 as an add-on to standard treatment improved cognition and general health in patients who had had an ischaemic stroke (16). An umbrella review evaluated the efficacy and safety of ischaemic stroke therapies. The results showed that *Ginkgo biloba* among other treatments can improve the neurological deficits and activities of daily living after ischaemic stroke (17). EGb 761 therefore appears to be a reasonable therapeutic approach for the treatment of cognitive impairment after ischaemic stroke. To gain further insight into its potential value in improving cognitive deficits and related conditions, we conducted a meta-analysis of pooled data from trials in dementia patients who had previously had a cerebral infarction confirmed by neuroimaging.

## 2 Methods

### 2.1 Literature search

A systematic literature search was performed in the databases Pubmed (PubMed nih.gov), EmBase, and the Cochrane Library (Search | Cochrane Library) in September 2024. The following terms and their variants were used as search terms: extract of *Ginkgo biloba*, Ginkgo leaf, dementia, Alzheimer’s disease (AD). No restrictions were placed on the languages of publication or the search period. Details of the search procedure are provided in the online Supplementary material.

From the search results, we selected randomised placebo-controlled trials with a daily dose of 240 mg EGb 761, (1) which included patients with VaD or Alzheimer’s disease (AD) with cerebrovascular disease, established by formal diagnostic criteria, (2) for which structured records of computed tomography (CT) or MRI findings were available to identify the presence of previous cerebral infarction, and (3) for which clinical outcome data were available.

### 2.2 Data extraction and quality assessment

Two reviewers independently reviewed the titles and abstracts and excluded irrelevant records. Then, the full text of the remaining records was read, and the final literature was selected for meta-analysis.

Quality assessment was carried out by calculating the Jadad score (18) to assess the risk of bias (Table 1). This is a simple scoring instrument consisting of 5 criteria that cover adequate randomization, adequate blinding, and thorough documentation of all patients enrolled in the study. Scores range from 0 (high risk of bias) to 5 (low risk of bias).

**Table 1:** Placebo-controlled trials assessing the efficacy of EGb 761 in dementia.

Clinical trials included in meta-analysis			
Trial	Publication	Jadad score	Patient population
1	Nikolova, 2013 (26)	5	NINCDS/ADRDA probable AD or NINDS/AIREN probable VaD or mixed form; SKT 9–23
2	Herrschaft, 2012 (29)	5	NINCDS/ADRDA probable AD or NINDS/AIREN probable VaD or mixed form; SKT 9–23
3	Ihl, 2011 (30)	5	NINCDS/ADRDA probable AD or NINDS/AIREN probable VaD or mixed form; SKT 9–23
4	Napryeyenko, 2007 (27)	5	NINCDS/ADRDA probable AD or NINDS/AIREN probable VaD or mixed form; SKT 9–23

Clinical trials not included in meta-analysis		
Trial	Publication	Reasons for exclusion from meta-analysis
5	McCarney et al. 2008 (44)	Diagnoses not made using formal criteria, influence of previous and concomitant medication unclear
6	Mazza et al. 2006 (45)	Treatment was not EGb 761
7	Schneider et al. 2005 (46)	No structured records of CT/MRI examination available
8	Le Bars et al. 2003^*^ (47)	No structured records of CT/MRI examination available, dosage not 240 mg EGb 761 daily
	Le Bars et al. 2000^*^ (43)	
9	van Dongen et al. 2003^*^ (48)	Uncertain diagnoses, most patients had age-associated mild cognitive impairment
	van Dongen et al. 2000^*^ (49)	
10	Kanowski, Hoerr 2003^*^ (50)	No structured records of CT/MRI examination available
	Kanowski et al. 1996^*^ (51)	
11	Maurer et al. 1997 (52)	No structured records of CT/MRI examination available
12	Hofferberth 1994 (53)	No structured records of CT/MRI examination available
13	Weitbrecht, Jansen 1986 (54)	No structured records of CT/MRI examination available, dosage not 240 mg EGb 761 daily

^*^Two publications reported primary data of the same trial.

NINCDS/ADRDA, National Institute of Neurological and Communicative Disorders and Stroke/Alzheimer's Disease and Related Disorders Association; NINDS/AIREN, National Institute of Neurological Disorders and Stroke/Association Internationale pour la Recherche et l’Enseignement en Neurosciences; SKT, Short Cognitive Performance Test.

### 2.3 Clinical outcomes

According to the revised ‘Guideline for the clinical investigation of drugs for Alzheimer’s disease’ (CPMP/EWP/553/95 Rev.2, 2018) issued by the European Medicines Agency (EMA), the primary domains for outcome assessment are cognition, activities of daily living (ADL) and global clinical status. Therefore, this meta-analysis was conducted for the following endpoints.

- **Change of cognition scores from baseline to end of treatment:**
  All studies enrolled patients from the outpatient clinics of psychiatric or neurological hospitals. The cognitive assessments were conducted at baseline, after 12 weeks, and at the final visit. All trials applied the Short Cognitive Performance Test (SKT) (19) as the outcome measure for cognition. The SKT is a simple, validated test for assessing cognitive impairment, predominantly of memory and attention, but also of executive function and word fluency (20). It consists of nine subtests, each with a performance time of 60 s at maximum. Thus, testing time is usually 10–15 min, and scoring and evaluation can also be quickly accomplished (19, 21). SKT scores and score changes correlate well with those of the Alzheimer’s Disease Assessment Scale—cognitive subscale and the Mini-Mental Status Examination (MMSE) (22) and with activity-of-daily-living scores as assessed by the ADL-IS (23). The SKT has been validated for the assessment of cognitive impairment in individuals with mild neurocognitive impairment and mild dementia (24); its cross-cultural validity has been demonstrated (25). The investigators underwent training in the administration process, which was conducted by an experienced geriatric psychiatrist utilising the original test materials.
- **Change in ADL scores from baseline to end of treatment:**
  Two trials (26, 27) applied the ADL subscale of the Gottfries–Bråne–Steen Scale (GBS) (28). The resulting score has a maximum of 36 with higher scores indicating more severe impairment. In the other trials (29, 30) the Activities of Daily Living-International Scale (ADL-IS) was used, ranging from 0 = no problems with activities of daily living to 4 = no longer performing any of the ADL-IS activities (31).
- **Change of the global assessment from baseline to end of treatment:**
  The global clinical condition was assessed using either the GBS Scale total score (28) or the Alzheimer’s Disease Cooperative Study Clinical Global Impression of Change (ADCS-CGIC) (32), respectively. The GBS scale is a tool for global assessment of dementia symptoms based on a semi-structured interview and observation of the patient. The scale measures intellectual, emotional, behavioural and psychological symptoms as well as activities of daily living. The ADCS-CGIC measures the global clinical rating of change from baseline with 1 = marked improvement over 4 = no change to 7 = marked worsening (32). Safety was assessed by physical examination, electrocardiography, and laboratory tests at screening and at the end of treatment after 22 or 24 weeks, respectively. Adverse events were recorded at all visits and during telephone calls at weeks 6 and 17 or 18. For pooled data, the number and relative frequency of subjects reporting at least one adverse event was analysed.

### 2.4 Statistical analysis

This meta-analysis of treatment effects of EGb 761 compared to placebo in patients with dementia following a cerebral infarction was based on individual patient data. The relevant data from all selected studies was provided by the sponsor of the analysed studies (Dr. Willmar Schwabe GmbH & Co. KG, Germany).

Demographic and baseline characteristics for the pooled data set were summarized using descriptive statistics. Categorical variables were presented as frequencies and percentages. For the continuous variables at baseline, mean, standard deviation (SD), median, and confidence intervals (CI) for the mean were calculated.

### 2.5 Handling of intercurrent events and missing data

A re-analysis of the therapeutic effects of EGb 761 was performed considering intercurrent events and applying the treatment policy strategy in the original study populations with the above mentioned endpoints. Study or treatment discontinuation for any reason and the use of prohibited concomitant medications were defined post-hoc as intercurrent events.

The last observation carried forward method used to replace missing values in the underlying studies was also substituted. Missing items of multi-item psychometric scales were imputed with available values if at least 50% of the item values were documented for the subject and visit. These imputations were made before calculating total scores or subscores of the scales. If more than 50% of individual items were missing, the total score, subscores or single item scores (endpoint values) were imputed, depending on the assumption of a monotone or non-monotone missing pattern. Missing data due to intercurrent events were imputed multiple times under either a non-random missing pattern assumption (using information from the placebo arm) or a random missing pattern assumption (using information from patients in the same treatment group).

### 2.6 Meta-analysis

Results for the patient subgroup with previous cerebral infarction in single trials were calculated using the SAS 9.4 software. The meta-analyses were performed with the R package meta (version 4.3-2, function metacont). Inverse variance weighting was applied for combining the results of the individual trials. As the focus was on the analysis of subgroups of the original trials, random-effects models were chosen for each outcome variable to reflect the potential heterogeneity that this might entail. Furthermore, it was regarded as a more conservative methodology in comparison to the application of fixed-effects models. Mean differences (MD) were estimated for the continuous outcomes when the same scale was used in all trials; standardized mean differences (SMDs) were calculated when different scales were used to measure the same outcome. An SMD of 0.2 was considered small, 0.5 medium, and 0.8 large. Statistical heterogeneity was determined using the between-study variance *τ*^2^, the *Q* statistic and the corresponding *p*-value. Statistically significant heterogeneity was assumed if the p-value was less than 0.1. In this case, a sensitivity analysis was performed by removing studies highly influencing the overall results.

Based on the imputed data of each individual trial, analyses of covariance with treatment group as factor and baseline outcome of each variable as a covariate were conducted for cognition and ADL outcomes. Where baseline scores were not available for the global impression outcomes, analysis of variance with a treatment factor was applied.

For the safety analysis, event risk differences and the odds ratios with 95% CIs were calculated for patients reporting adverse events or suspected adverse drug reactions. Given the conservative approach taken throughout the analysis, fixed-effects models were employed for comparing the treatment groups.

## 3 Results

### 3.1 Selection of studies

**Figure 1:**
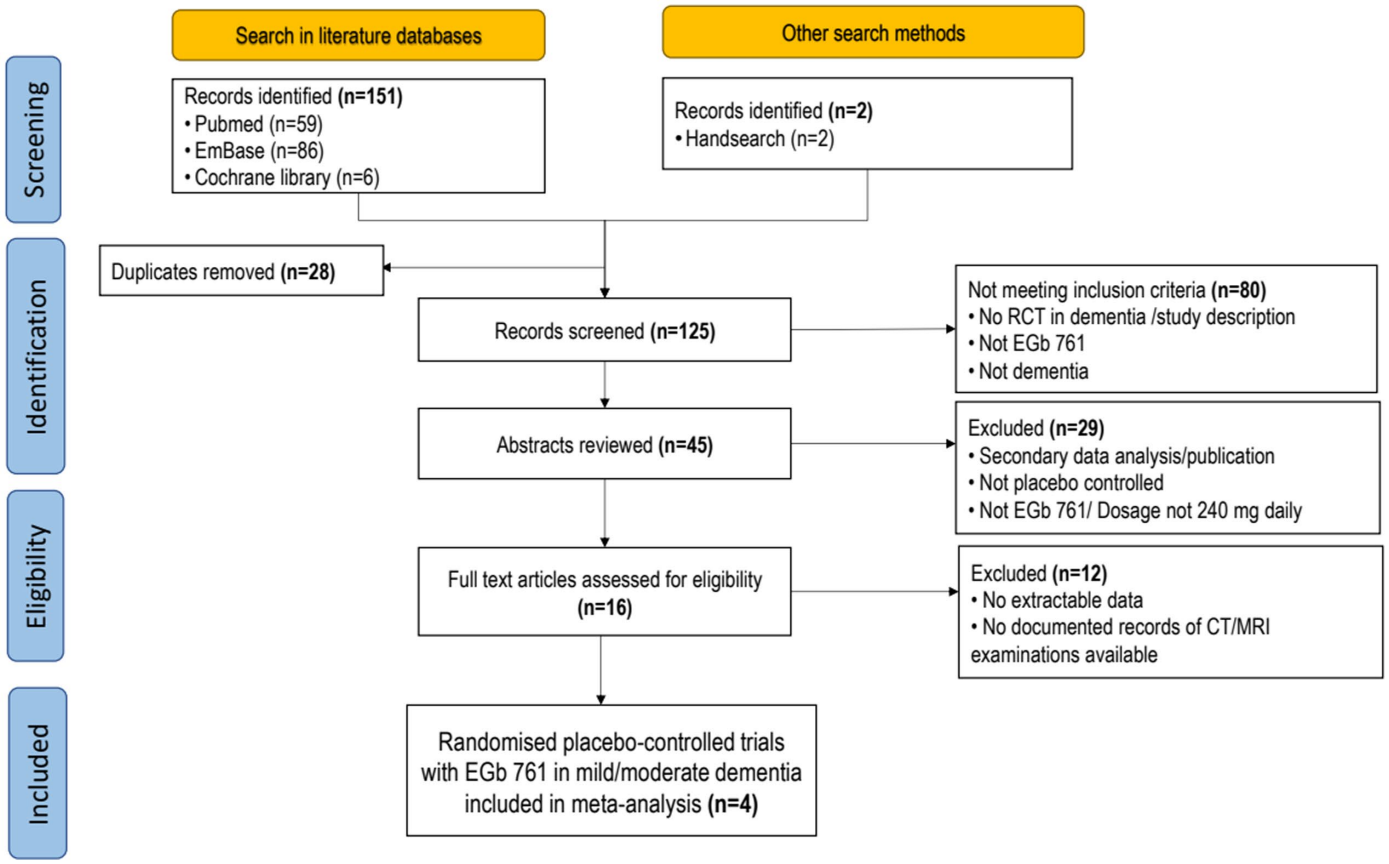
PRISMA flow chart.

The results of the search and selection procedure are depicted in Figure 1. Four randomised placebo-controlled, double-blind trials were selected for this meta-analysis (Table 1). All of them were sponsored by Dr. Willmar Schwabe GmbH & Co. KG Pharmaceuticals, Karlsruhe, Germany. Three trials were originally published in English. One was published in Bulgarian, and a translation was created by a qualified professional.

All four studies investigated the efficacy of EGb 761 in patients with mild to moderate dementia (total score 9–22 on the SKT). In each study, a daily dose of 240 mg EGb 761 was taken for 22 or 24 weeks. Patients diagnosed with probable VaD (33) or possible AD with cerebrovascular disease (33, 34), respectively, who had previously experienced a cerebral infarction as confirmed by neuroimaging results, were selected from both the EGb 761 and placebo groups. CT or MRI scans had been performed at the screening visits or within 12 months prior to study enrolment. The images were assessed by radiologists; the diagnoses were established by the attending physicians and documented in the patients’ medical records.

### 3.2 Patient characteristics

Of the 1,608 patients in the four trials analysed, 488 patients (full analysis set, FAS) were identified with evidence of cerebral infarction on CT or MRI: 241 in the EGb 761 treatment group and 247 in the placebo group. An overview of patient characteristics is shown in Table 2. There were more women in both groups, accounting for around 60%. The mean age of patients was 68 years (EGb 761 group) and 66 years (placebo group), respectively.

**Table 2:** Baseline characteristics of patients with previous cerebral infarction in both treatment arms.

Clinical trial	Treatment group	No. of patients with cerebral infarction		Age (years)	Gender (% of patients)	
		Safety set (randomised/(drop-out: *n*[%]))	FAS	Mean (SD)	Female	Male
Nikolova, 2013 (26)	EGb 761	78 (7 [9.0%])	73	71 (8)	39.7	60.3
	Placebo	77 (6 [7.8%])	76	69 (8)	52.6	47.4
Herrschaft, 2012 (29)	EGb 761	49 (3 [6.1%])	46	66 (9)	67.4	32.6
	Placebo	52 (3 [5.8%])	51	67 (10)	60.8	39.2
Ihl, 2011 (30)	EGb 761	58 (5 [8.6%])	57	69 (10)	63.2	36.8
	Placebo	57 (1 [1.8%])	57	68 (9)	59.6	40.4
Napryeyenko, 2007 (27)	EGb 761	66 (2 [3.0%])	65	65 (7)	69.2	30.8
	Placebo	65 (2 [3.1%])	63	64 (8)	77.8	22.2
Total	EGb 761	251 (17 [6.8%])	241	68 (9)	58.5	41.5
	Placebo	251 (12 [4.8%])	247	66 (9)	62.3	37.7

FAS, Full analysis set; SD, Standard deviation.

### 3.3 Outcome variables at baseline

The baseline values of the outcome variables are shown in Table 3. In all trials, the treatment groups had similar baseline values for the outcome variables. The degree of cognitive impairment was similar across the four trials.

**Table 3:** Baseline values of outcome variables for subgroups of patients with dementia after cerebral infarction (FAS).

Clinical trial	Treatment group	No. of patients	Cognition (SKT)	ADL^1^	Global assessment^2,3^
			Mean (SD)	Mean (SD)	Mean (SD)
Nikolova, 2013 (26)	EGb 761	73	15.2 (4.2)	3.3 (4.3)	35.2 (6.8)
	Placebo	76	15.6 (3.9)	3.1 (4.0)	35.1 (8.8)
Herrschaft, 2012 (29)	EGb 761	46	14.7 (4.4)	1.7 (0.6)	n.a.
	Placebo	51	15.5 (4.2)	1.8 (0.6)	n.a.
Ihl, 2011 (30)	EGb 761	57	18.2 (3.8)	2.0 (0.7)	n.a.
	Placebo	57	18.5 (3.6)	2.1 (0.5)	n.a.
Napryeyenko, 2007 (27)	EGb 761	65	14.7 (4.0)	6.4 (3.8)	35.2 (6.8)
	Placebo	63	15.0 (3.8)	6.4 (4.1)	35.1 (8.8)

SD, Standard deviation; n.a., not applicable; SKT, Short Cognitive Performance Test; ADL, activities of daily living.

^1^Ihl, 2011 and Herrschaft, 2012 used the Alzheimer’s Disease Activities of Daily Living-International Scale (ADL-IS), Nikolova, 2013 and Napryeyenko, 2007 used the activities of daily living subscale of the Gottfries–Bråne–Steen Scale (GBS ADL).

^2^Ihl, 2011 and Herrschaft, 2012 used the Clinical Global Impression of Change adapted by the Alzheimer’s Disease Cooperative Study (ADCS-CGIC); Nikolova, 2013 and Napryeyenko, 2007 used the Gottfries–Bråne–Steen Scale (GBS) total score.

^3^Since the ADCS-CGIC assesses the change from baseline, no baseline values were available.

### 3.4 Results of treatment group comparisons

**Figure 2:**
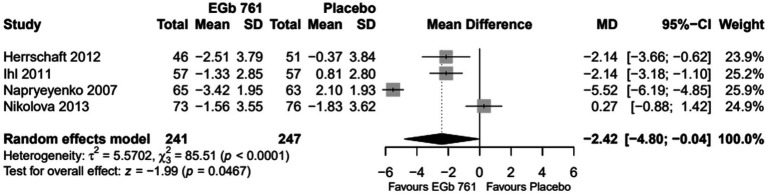
Change scores for cognition (SKT) in patients with previous cerebral infarction—all trials (FAS, placebo-based imputation).

**Figure 3:**
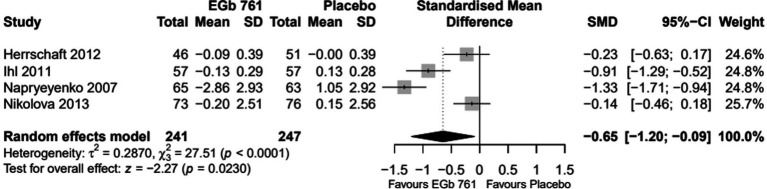
Change scores for activities of daily living (Activities of daily living subscale of the Gottfries–Bråne–Steen Scale, Alzheimer’s disease activities of daily living international scale) in patients with previous cerebral infarction—all trials (Full Analysis Set, placebo-based imputation).

**Figure 4:**
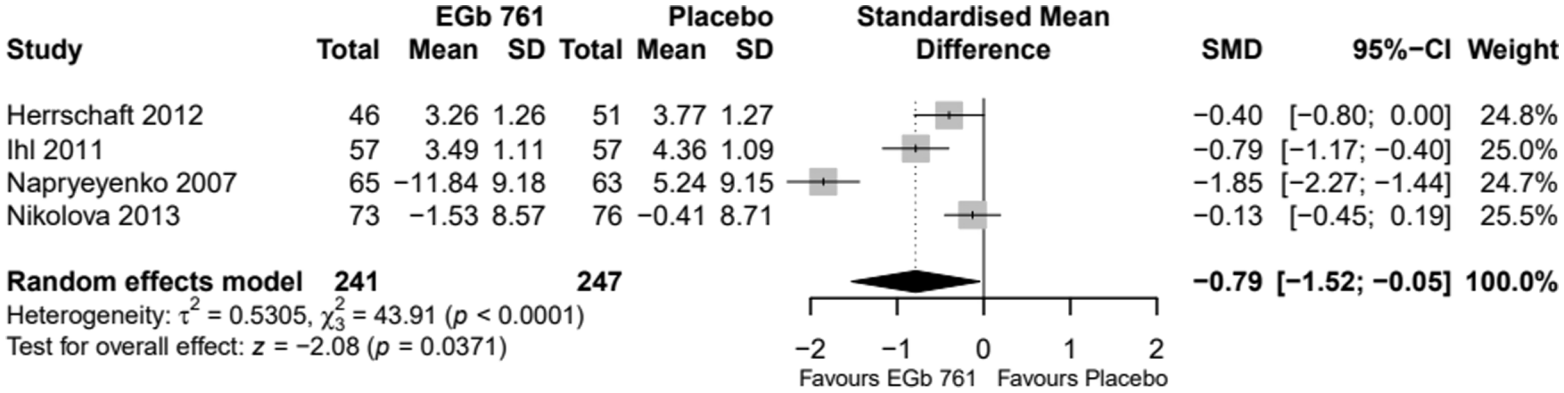
Change of scores for global assessment (Gottfries–Bråne–Steen scale, clinical global impression of change adapted by the Alzheimer’s disease cooperative study) in patients with previous cerebral infarction—all trials (FAS, placebo-based imputation).

The results of the meta-analyses for each outcome variable are depicted in Figures 2–4.

#### 3.4.1 Cognition

The change in SKT total score ranged from −3.4 to −1.3 in the EGb 761 groups and from −1.8 to 2.1 in the placebo groups. In three of the four trials (27, 29, 30), patients treated with EGb 761 showed a statistically greater improvement in cognition compared to the placebo group. The meta-analysis showed a statistically significant mean difference (MD) in favour of EGb 761 for the random effects model (MD = −2.42, 95% CI [−4.80; −0.04], *p* = 0.0467, Figure 2).

#### 3.4.2 Activities of daily living

Standardized mean differences (SMD) were applied to compare across studies since two of the trials used the GBS-ADL subscale (26, 27) and two used the ADL-IS (29, 30) to assess changes in activities of daily living. The overall difference was statistically significant in favour of EGb 761 (SMD = −0.65 [−1.20; −0.09], *p* = 0.0230, Figure 3).

#### 3.4.3 Global assessment

The change of the clinician’s global assessment was evaluated as SMD in the meta-analysis because two different scales were applied in the trials (ADCS-CGIC and the GBS total score). The difference between the treatment groups was statistically significant in the pooled analysis (SMD = −0.79 [−1.52; −0.05], *p* = 0.0371, Figure 4).

#### 3.4.4 Sensitivity analysis

Statistically significant heterogeneity was observed across all outcomes, which was mainly related to two trials (26, 27). Therefore, an additional sensitivity analysis was carried out by excluding both studies (26, 27). Results of this sensitivity analysis showed a reduction of heterogeneity (e.g., for cognition *τ*^2^ = 0, MD = 2.14 [−3.00; 1.28], *p* < 0.0001, *N* = 2).

### 3.5 Safety

There were 152 adverse events and 10 serious adverse events in 251 patients treated with EGb 761 (safety analysis set), compared to 151 adverse events and 11 serious adverse events in 251 patients who took placebo. This resulted in a risk difference of 0.2% [95% CI: −7.67, 8.01%] for all adverse events, or an odds ratio of 1.01 [95% CI: 0.68, 1.49]. All serious adverse events were assessed by the investigators as being not related to the study drug.

Physical and neurological examinations, 12-lead electrocardiogram, blood pressure, heart rate, and laboratory tests performed before the start of treatment and at the end of the study did not reveal any clinically relevant or systematic changes with EGb 761.

## 4 Discussion

This meta-analysis was conducted using pooled data from subgroups of patients with mild to moderate dementia who had a previous cerebral infarction detected by neuroimaging. We did not rely on reports of clinical stroke in the patients’ medical history, because such events may not be remembered reliably by patients with dementia or caregivers who had not lived with the patients earlier. All studies included patients with mild to moderate dementia, either due to AD, VaD, or mixed pathology. The results showed that patients treated with EGb 761 improved significantly more than those in the placebo group in the areas of cognition, activities of daily living and global assessment. Rates of adverse events and serious adverse events were not significantly different between EGb 761 and placebo. Of note, the individual studies were powered to show statistically significant treatment effects in the overall population. Cerebral infarctions were documented in approximately 30% of the total population. Nevertheless, statistically significant differences were demonstrated, resulting in SMDs above 0.6, which can be classified as moderate effect sizes (35). Our meta-analysis confirmed significant improvements in both co-primary endpoints, i.e., in activities of daily living and in the global endpoints and the effect of EGb 761 on cognition may therefore be considered clinically meaningful in terms of current guidelines (CPMP/EWP/553/95 Rev.2, 2018).

EGb 761 extract is a complex mixture of plant compounds that exhibit diverse pharmacological activities on the multifactorial pathophysiology of neurodegenerative conditions (36). Treatment with EGb 761 has been shown to reduce neuroinflammation, a central contributor to the disease. Further disease mechanisms include neurodegeneration, mitochondrial damage, oxidative stress and dysfunction of the microbiota-gut-brain axis. EGb 761 can stimulate neuroplasticity, protect and restore mitochondrial function, reduce the formation of p-tau and amyloid *β* plaques, act as an antioxidant, improve cerebral blood flow and restore a dysfunctional microbiome (36).

Nevertheless, the complex mechanism of action of EGb 761 in relation to neurodegenerative processes has not yet been fully elucidated. This is partly due to general limitations of pharmacological studies related to complex and multifactorial pathways. At the same time, the general lack of mechanistic understanding of its efficacy is a limitation of this meta-analysis. In the future, modern bioinformatics methods will probably allow to close this gap. These tools may predict the physicochemical, pharmacokinetic, and toxicological properties of plant compounds identified through chemical analysis. Network pharmacology enables the mapping of signalling pathways involved in the pathogenesis of cognitive decline or dementia, providing a deeper understanding of the mechanisms of action. This approach has already been successfully applied to the research of other plant-based medicines (37, 38). The results and effect sizes determined by the present analyses are consistent with those reported in a meta-analysis conducted in the diagnostic subgroups of probable AD, probable VaD, and mixed dementia (39). In all subgroups analysed in this former meta-analysis, significant superiority of EGb 761 over placebo was observed for all outcome measures, except for quality of life (QoL) (39). However, the QoL assessment may have lacked statistical power due to the small size of the subsample. Furthermore, other confounding factors, such as stroke-related neurological deficits, may have been involved.

In all studies included in this meta-analysis, the SKT was employed as an outcome measure for cognition. Based on the experience with 1,624 elderly patients in Shanghai, this test had been demonstrated to exhibit good stability and may therefore be considered a reliable and valid screening tool for detecting mild cognitive impairment (25).

The findings of our meta-analysis align with those of a previous clinical study which enrolled patients within seven to 14 days post stroke (16). Following a 24-week course of treatment, EGb 761 was observed to elicit improvements in global cognitive performance, as evidenced by a more pronounced increase in the MoCA score. Additionally, the treatment group demonstrated enhanced memory function, as indicated by a greater improvement in the delayed recall scores of the MoCA and the Hopkins Verbal Learning Test compared to the control group (16).

Patients included in our meta-analysis were diagnosed with VaD or AD with cerebrovascular disease. Additionally, they had a history of cerebral infarction as evidenced by neuroimaging. It can therefore be concluded that they suffered from a form of probable major vascular cognitive impairment according to the definition of the Vascular Impairment of Cognition Classification Consensus Study group (40).

Heterogeneity across all outcomes was mainly related to two trials (26, 27). In one of them, the observed differences between treatment and placebo groups were not statistically significant, although the results demonstrated a favourable outcome for EGb 761 (26). The patients in the study exhibited a less severe pathology. Furthermore, notable improvements were observed in the placebo group of this trial, which may have been due to improved overall patient care over the course of the study. The other trial showed unexpectedly large effects (27). The particularly favourable overall results are probably related to the greater net benefit of EGb 761 treatment in patients with neuropsychiatric symptoms. These were clearly recognizable in this study by the reported mean Neuropsychiatric Inventory score of 21 (41). The presence of high heterogeneity has the potential to compromise the certainty of the evidence and restrict the generalisability of the outcomes. This poses a significant challenge to clinicians attempting to integrate results into individual patient care. However, a sensitivity analysis was conducted, which excluded trials that caused heterogeneity, i.e., those with the largest effects (27), and those with the smallest effects (26). This reduced the heterogeneity and confirmed the results of the meta-analyses calculated across all four trials.

The selection of studies was limited to a daily dose of 240 mg of EGb 761 because this dosage has been shown to stabilize cognition, function, behaviour, and global change (42). It appears that the 120 mg daily dose may have been insufficient, although in some studies this dosage was shown to be effective (43). This fact indicates possible a dose–response relationship. As no structured CT or MRI data were available from the studies employing a daily dosage of 120 mg, these studies could not be included in the meta-analysis. Consequently, it was neither possible to include randomised controlled trials across various dosage levels nor to further investigate the impact of dose on the outcomes.

As a post-hoc analysis, the present study is susceptible to the potential influence of selection as well as publication bias. The generalisability of the results of this meta-analysis is further limited by the potential influence of sponsorship bias, given that all studies were sponsored by the manufacturer of EGb 761. It is therefore recommended that further research will be conducted to confirm these findings. Although patient characteristics were balanced in both groups, the absence of an evaluation of stroke severity in the studies precludes an assessment of this variable in the present analysis. This is a limitation of the study, as stroke severity is a well-established risk factor for cognitive impairment. Same applies to the lack of information on previous or concurrent treatments for stroke or prevention of recurrent stroke. The present study has revealed no evidence to suggest that the percentage of patients receiving guideline-directed treatment, or possibly being undertreated, differs between the treatment groups. Moreover, potential confounding due to the types and combinations of vascular risk factors and comorbidities was not considered. There is, however, no indication of an influence of such factors on the efficacy of EGb 761 in patients with dementia. The results of this meta-analysis must be interpreted with these limitations in mind.

## 5 Conclusion

In conclusion, the meta-analysis of pooled data from four clinical trials indicates efficacy of a daily 240 mg dose of EGb 761 for patients with dementia resulting from a cerebral infarction or probable major vascular cognitive impairment. However, hard evidence is still lacking and would require confirmation in dedicated clinical trials. If future mechanistic approaches based on chemical analysis, bioinformatics and molecular biology allow to fully elucidate the mechanism of action, this will reinforce the results further.

## Data Availability

The original contributions presented in the study are included in the article/Supplementary material, further inquiries can be directed to the corresponding author.

## Footnotes

1 EGb 761® is the proprietary extract and active ingredient in pharmaceuticals such as Ginaton manufactured by Dr. Willmar Schwabe, Karlsruhe, Germany.
