# Supplementary material for: Ginkgo biloba extract EGb 761 in patients with dementia and a history of cerebral infarction—meta-analysis of pooled data from randomised clinical trials
Source: Front Neurol. 2026 Mar 13;16:1658064. doi: 10.3389/fneur.2025.1658064 (PMC13023401; doi:10.3389/fneur.2025.1658064)
Supplement: Supplementary file 1 [file Data_Sheet_1.pdf]

Supplemental online material

Search strategy, search performed on September 3, 2024

Pubmed (PubMed (nih.gov))

Step	Search terms	No. of hits
#1	Dementia[MeSH]	218,358
#2	Dementia*[Title/Abstract]	155,689
#3	((((((((((((((((“ginkgo biloba”[Title/Abstract]) OR (“ginkgo biloba 761”[Title/Abstract])) OR (“ginkgo biloba egb”[Title/Abstract])) OR (“ginkgo biloba egb 761”[Title/Abstract])) OR (“ginkgo biloba extract egb 761”[Title/Abstract])) OR (“ginkgo biloba extract gbe”[Title/Abstract])) OR (“gingko biloba extract”[Title/Abstract])) OR (“gingko biloba extract egb”[Title/Abstract])) OR (“gingko biloba extract egb 761”[Title/Abstract])) OR (“maidenhair extract”[Title/Abstract])) OR (“maidenhair tree”[Title/Abstract])) OR (“egb 761”[Title/Abstract])) OR (“gbe 761”[Title/Abstract])) OR (tebonin[Title/Abstract])) OR (tanakan[Title/Abstract])) OR (“rokan”[Title/Abstract])) OR (“ginkoba”[Title/Abstract])	4,655
#4	#1 or #2	278,603
#5	#3 and #4	477
#6	(“randomized controlled trial”[Publication Type]) OR (“controlled clinical trial”[Publication Type])	712,341
#7	#5 an #6	58

EmBase via CAS STNext®

Step	Search string	No. of hits
L1	(DEMENTIA* OR ALZHEIMER*)/TI	201,429
L2	(“GINKGO BILOBA” OR “GINKGO BILOBA 761” OR “GINKGO BILOBA EGB” OR “GINKGO BILOBA EGB 761” OR “GINKGO BILOBA EXTRACT EGB 761” OR “GINKGO BILOBA EXTRACT GBE”)/TI,AB	5,820
L3	(“GINGKO BILOBA EXTRACT” OR “GINGKO BILOBA EXTRACT EGB” OR “GINGKO BILOBA EXTRACT EGB 761” OR “MAIDENHAIR EXTRACT” OR “MAIDENHAIR TREE” OR “EGB 761” OR “GBE 761” OR TEBONIN OR TANAKAN OR “ROKAN” OR “GINKOBA”)/TI,AB	1,125
L4	L2 OR L3	6,105
L5	L1 AND L4	378
L6	L5 AND “RANDOMIZED CONTROLLED TRIAL? “ OR “CONTROLLED CLINICAL TRIAL?”	125
L7	L6 NOT CONFERENCE/DT	117
L8	L7 NOT “SYSTEMATIC REVIEW?”/DT	114
L9	L8 NOT “SYSTEMATIC REVIEW?”	86

Cochrane library (Search | Cochrane Library)

Step	Search string	No. of hits
#1	(“Ginkgo biloba”):ti,ab,kw AND (“placebo-controlled study”):ti,ab,kw AND (“dementia”):ti,ab,kw	6
